# Emerging Longitudinal Trends in Health Indicators for Rural Residents Participating in a Diabetes and Cardiovascular Screening Program in Northern Alberta, Canada

**DOI:** 10.1155/2011/596475

**Published:** 2011-04-27

**Authors:** Kelli Ralph-Campbell, Richard T. Oster, Tracy Connor, Ellen L. Toth

**Affiliations:** BRAID Research Group, University of Alberta, 8308 114 Street, Suite 1055, Edmonton, AB, Canada T6G 2V2

## Abstract

*Background*. Geographic isolation, poverty, and loss of culture/tradition contribute to “epidemic” rates of diabetes amongst indigenous Canadians. The Mobile Diabetes Screening Initiative travels to rural indigenous and other remote communities in Alberta to screen for diabetes and cardiovascular risk. We sought to examine risk factors longitudinally. *Methods*. Clinical and anthropometric measurements were undertaken for 809 adults (aged 20–91) between November 2003 and December 2009. For those who had more than one MDSi visit, trend estimates (actual changes) were calculated for body mass index (BMI), weight, waist circumference, hemoglobin A1c (A1c), total cholesterol, and blood pressure. *Results*. Among those without diabetes (*N* = 629), BMI and weight increased (*P* < .01) and blood pressure decreased (*P* < .05). For those with diabetes (*N* = 180), significant improvements (*P* < .05) were observed for all indicators except waist circumference. *Conclusion*. Improvements observed suggest that MDSi's model may effectively mediate some barriers and support subjects in managing their health.

## 1. Introduction

In 2006-2007, approximately two million Canadians (6% of the country's population) had diagnosed diabetes [1]. This rate is projected to increase to 2.6 million by 2011, representing a 33% increase from 2006 [2, 3].

The International Diabetes Federation (IDF) estimates about half of diabetes cases remain undiagnosed and, therefore, estimated Canada's national prevalence for people aged 20–91 to be 9% for 2007, and 12% for 2010 [4]. Canada ranks among the world's industrialized countries with the highest rates of type 2 diabetes. Add to this an impaired glucose tolerance (IGT) prevalence estimated at 13% for Canada for 2010 [4]. Diabetes-related costs consumed an estimated 13% of total health care spending in Canada in 2010 [4].

Increases in prevalence are expected to continue as Canada's demographic profile shifts with the aging population, increased immigration from high-risk populations, and a growing indigenous population [5]. Age-adjusted prevalence rates have been estimated to be 3.6 times higher for First Nations indigenous men and 5.3 times higher for First Nations indigenous women compared to the general Canadian population [6]. 

In addition to First Nations people, Canada recognizes two other indigenous groups: the Inuit, inhabitants of the far North, and the Métis, who are children of relationships between Europeans and the indigenous population prior to and throughout the colonial settlement of Canada. These relationships established political alliances and provided the colonial regimes with local expertise regarding subsistence and harvesting natural resources [7]. The Métis are distinct from First Nations populations in terms of culture, history, and sociopolitical and socioeconomic factors. 

Little is known about diabetes prevalence among the Inuit or the Métis people in Canada though in the 2001 Aboriginal Peoples Survey (APS), 6% of Métis respondents reported having diabetes [8]. In Alberta self-reported prevalence of diabetes was 6% in 2006, with a crude diabetes prevalence increase of 66% between 1998 and 2006 [9].

Located in Western Canada, the province of Alberta stretches 1,223 kilometres from north to south, 660 kilometers east to west (661,848 square kilometers). Much of northern Alberta is isolated, covered by boreal forest, with a subarctic climate. Adequate road access is an issue for many rural communities, especially those in the north. 

Alberta has an ethnically diverse population of 3.7 million people (2009), of which about 6% self-identifies as indigenous (of Métis, First Nations, or Inuit heritage) [10]. Eighty-one percent of the province's total population resides in metropolitan and urban areas; 19% live in rural areas [11]. In 2009, 63% of Alberta's indigenous population resided in urban areas [12].

In 2009, 85,500 Métis people lived in Alberta. Eighty-eight percent resided in major urban centres, while 7,990 (approximately 10%) were members of eight incorporated Métis settlements which have unique terms of governmental jurisdiction for service delivery [12, 13]. 

In 2009, there were 105,301 First Nations people residing in Alberta, of whom 36% lived off reserve [12].

The province's three major metropolitan centres are located in central and southern Alberta; in northern Alberta, there are two major urban centres—Grande Prairie, population 50,227; Fort McMurray, population 63,676—and only two towns (Slave Lake and Peace River) with populations over 5,000 [10]. Many northern residents find employment at industry sites centralized in the north, such as the Athabasca Oil Sands.

Generalists (general practitioners and family doctors) provide the majority of medical care for Albertans with diabetes, with rural residents having lower rates of specialist care visits compared to metropolitan residents [2]. The increase in diabetes prevalence has meant increased demand for health care resources, particularly to address comorbid conditions and the complications of diabetes [2]. Findings in the 2009 Alberta Diabetes Atlas make clear the need for enhancing primary prevention and management, such as ensuring adequate numbers of generalist practitioners and allied health professionals (nurses, dietitians, and pharmacists). Additionally, culturally appropriate prevention and management initiatives should be prioritized for indigenous people and communities [2].

In 2003, Alberta initiated a 10-year diabetes strategy, through which the province has funded the Mobile Diabetes Screening Initiative (MDSi). MDSi operates out of the University of Alberta in Edmonton and is a collaborative effort between university researchers, community stakeholders, Alberta Health and Wellness, northern zone service providers, and policy/decision-maker stakeholders. MDSi travels to 25 Métis, off-reserve First Nations communities and other remote, rural communities in northern Alberta. The program provides screening services for diabetes, diabetes risk, and cardiovascular risk in community settings. (Most First Nations people in rural Alberta live on reserves, which are federal in primary health care jurisdiction, and as such, a similar but not identical program to MDSi carries out diabetes screening on reserves, under the auspices of the Aboriginal Diabetes Initiative [14, 15]). The program provides screening services for diabetes, diabetes risk, and cardiovascular risk in community settings.

Prevalence estimates of undiagnosed diabetes and diabetes and cardiac risk factors from MDSi screening have been reported elsewhere [9]. The objective of the present study was to undertake preliminary longitudinal analyses of MDSi data collected from 2003 to 2009, to identify trends (actual changes) in health indicators over time for subjects who had more than one visit during this period.

## 2. Methods

Two vans transported portable diagnostic equipment and a team of health professionals (nurse, dietitians, and lab technician) to the communities to provide diabetes and cardiovascular screening. The team usually had indigenous members, and for nonindigenous members significant and ongoing cultural awareness training was provided. 

Subjects were volunteers and enrolled in MDSi through self-referral in response to local promotion and outreach carried out in advance of MDSi visits. Adults as well as children were enrolled. Trend estimates for children will be reported elsewhere. Our current analyses include adults only (ages 20–91) with more than one MDSi visit. “Known” diabetes (i.e., presence of diabetes prior to their first MDSi visit) was confirmed by medications, chart review, or nurse history. 

Clinical and anthropometric measurements performed included height and weight (for calculating body mass index: BMI), waist circumference, blood pressure, blood glucose, lipids (triglycerides and total/fractionated cholesterol), and hemoglobin A1c (A1c). 

Subjects were asked to remove their shoes and any clothing (such as coats) to be weighed, and waist circumference was measured using a standard measuring tape at the iliac crest. Subjects rested for five minutes prior to a single, seated blood pressure reading.

Overweight (BMI 25–29.9) and obesity (BMI ≥ 30) were defined according to criteria from the National Cholesterol Education Program Adult Treatment Panel III (NCEP ATP III) [16]. An indigenous-specific definition does not exist for the metabolic syndrome; however, NCEP ATP III criteria has been shown to be a useful tool for evaluating diabetes risk in American Indians [17]. We, therefore, used the NCEP ATP III definition, for which the presence of three or more of the following are necessary for a diagnosis: increased waist circumference (>102 cm for males; >88 cm for females), elevated triglycerides (≥1.69 mmol/L), low HDL (high density lipoprotein) cholesterol (<1.0 mmol/L in males; <1.3 mmol/L in females), diabetes or impaired fasting glucose (IFG) (≥6.1 mmol/L), or hypertension (≥130/85 mmHg) [16].

Subjects had been asked to come to their appointment fasting though nonfasting subjects were accepted and tested. Blood was collected via a single finger puncture with the Accu-Chek Safe-T-Pro (Roche Diagnostics) lancet. The first blood droplet was discarded using a sterile cotton swab, and the subsequent blood was collected and immediately analyzed. 

Glucose and lipids were analyzed using the Cholestech L.D.X (Cholestech Corporation) portable analyzer, and A1c was analyzed using the Bayer DCA2000+ analyzer (Bayer Diagnostics). The Cholestech L.D.X utilizes both enzymatic methodology and solid-phase technology, whereas the Bayer DCA2000+ is an immunoassay. Performance assessments of both analyzers were provided by the Canadian External Quality Assessment Laboratory (CEQAL) with sample sets covering the clinical range of interest and accuracy target values assigned by credentialed reference methods. The base of accuracy for A1c was the Diabetes Control and Complications Trial (DCCT) Reference Laboratory at the University of Missouri, whereas CEQAL's Reference Method Laboratory was the base of accuracy for lipid measurements. MDSi field staff undertook day-to-day monitoring of the analytical performance of both instruments via an internal quality control program with predefined performance limits and accuracy targets assigned by reference methods. 

Adhering to the cutoffs set by the Canadian Diabetes Association (CDA), we defined undiagnosed diabetes as a fasting plasma glucose (FPG) ≥7.0 mmol/L and prediabetes as a FPG 6.1–6.9 mmol/L [5]. Since a single FPG sample is not adequate to confirm a diagnosis [5], our results only suggested a provisional diagnosis.

Subjects received a copy of their test results at the time of service delivery, and the MDSi nurse/dietitians provided subjects with individualized and culturally sensitive counseling based on their test results. Test results were entered into MDSi's proprietary clinical database, and subjects' prior screening results were available for reference during counseling. Test results were sent to subjects' primary care provider and discussed with local health care staff if available. 

MDSi staff additionally facilitated “lunch and learn” sessions to share knowledge with local care providers on such topics as appropriate screening tests, management strategies, and lifestyle challenges. 

The MDSi project was approved by the Health Research Ethics Board at the University of Alberta. Consent for aggregate analysis was obtained from subjects verbally and in writing; the consent form was read aloud by an MDSi field staff member prior to the subject signing. 

Individuals with and without known diabetes were analyzed separately. Mean baseline indicators were calculated. Mean baseline and subsequent indicators were also compared for individuals over time. 

Statistical analyses were done with SAS 9.1 (SAS Institute Inc., Cary, NC) and SPSS 17.0 (SPSS Inc., Chicago, IL). Trend estimates (actual changes) for each indicator were considered significant if *P* < .05. Standard deviations and 95% confidence intervals were determined for mean and prevalence values accordingly. For continuous variables, we used a logistic regression model, where the variables included covariates.

## 3. Results

A total of 2,954 unique subjects were screened between November 2003 and December 2009, and a total of 4,655 visits were completed (including repeat visits). Eight hundred and nine adults (aged ≥20) had repeat visits, typically at one year, for a return rate of 27%. Of these 809 individuals, 180 (66% Métis, 15% First Nations, and 19% nonindigenous) were identified with known diabetes and 629 (66% Métis, 17% First Nations, and 17% nonindigenous) without known diabetes. 

Subjects ranged from 20 to 91 years of age (median 45). We identified high baseline rates of overweight and obesity (87%), abnormal waist circumference (79%), hypertension (32%), and hypercholesterolemia (34%). See Table 1.

Of those subjects with known diabetes, the mean duration of diabetes was 8.4 years, and 50% had poor control as defined by Canadian Diabetes Association (CDA) criteria [5]. Of those without diabetes, approximately 2% had A1c >7.0% indicating undiagnosed diabetes.

**Figure 1:**
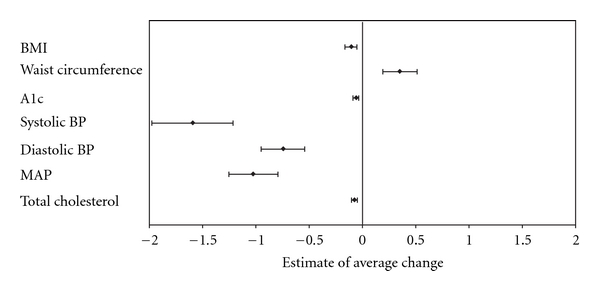
Trends for health indicators of returning subjects with known diabetes (*N* = 180). Values are estimates for the average actual change per year with 95% CI.

**Figure 2:**
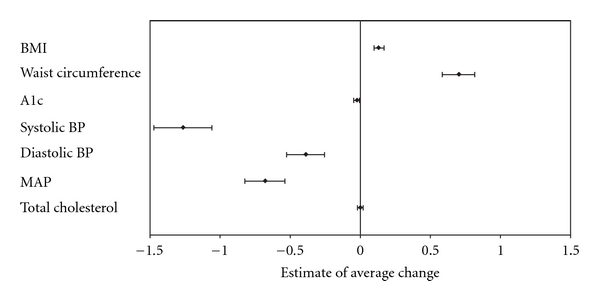
Trends for health indicators of returning subjects without known diabetes (*N* = 629). Values are estimates for the average actual change per year with 95% CI.

Overall trend estimates (actual changes) over time for adults are shown in Figures 1 and 2. For returning subjects with known diabetes, significant improvements (*P* < .05) were observed in BMI, blood pressure, total cholesterol, and A1c concentrations (Figure 1). Among returning subjects without known diabetes, improvement was only observed in blood pressure (*P* < .05), while BMI and waist circumference increased over time (*P* < .01) (Figure 2).

## 4. Discussion

Loss of culture, language, and traditional food, geographic isolation, poverty, and status/identity issues are some contributors to the “epidemic” rates of diabetes amongst Canada's indigenous population. Sadly, a systematic process of assimilation through forced attendance at residential schools resulted in serious sexual, physical and emotional abuses with consequences that resonate to this day and which have been the subject of an official apology by Canada's Prime Minister on 11 June 2008 [7, 18].

The MDSi model incorporates principles of participatory research—the ideal is equal participation in decision making shared between community stakeholders and researchers [19]—aimed at addressing some of these social determinants of health via a service delivery framework that is largely community based. While the CDA's 2003 Clinical Practice Guidelines (CPGs) recommended screening programs amongst indigenous Canadians be undertaken within the community setting [20], this recommendation was unfortunately excluded from the updated CPGs in 2008 [5].

The observed improvements amongst adults with known diabetes imply that MDSi's care model may play a role in supporting subjects to improve their health. However, since no control group was included, MDSi's contribution cannot be quantitated. Additionally, subjects were volunteer participants, which was likely a source of participant bias. Secular improvements in diabetes health may be due to a combination of effects including the availability of clinical practice guidelines and the federal Aboriginal Diabetes Initiative (ADI).

Our results for subjects with diabetes parallel trends reported from a similar project—SLICK (Screening for Limbs, I-eyes, Cardiovascular and Kidney complications of diabetes), funded by ADI—involving First Nations people with diabetes living on reserve in Alberta [21]. SLICK identified high baseline rates of overweight and obesity (92%), abnormal waist circumference (85%), poor and inadequate glucose control (60% and 35%), hypercholesterolemia (44%), and hypertension (62%). However, significant improvements in BMI, blood pressure, total cholesterol and HbA1c concentrations were seen over time for SLICK subjects who had repeat visits (typically at around one year) [21].

Longitudinal studies of administrative data in Canada and the United States have shown similar trends [22, 23]. 

Dannenbaum et al. showed that the proportion of Eeyou Istchee First Nations (Quebec) individuals with diabetes achieving HbA1c concentrations <7% significantly improved from 2002 to 2005 [22]. 

In the US, the Indian Health Service (IHS) reported significant improvements in HbA1c, cholesterol concentrations, and blood pressure amongst American Indians and Alaska Natives with diabetes from 1995 to 2001 [23, 24]. Additionally, in 1997, the US Congress established funding for the IHS Special Diabetes Program for Indians (SDPI), committing $150 million per year towards diabetes treatment and prevention in 399 IHS, Tribal, and Urban Indian health programs. In SDPI's first eight operational years, reported clinical outcomes for people with diabetes included significant reductions in mean HbA1c (8.9 to 7.9 mmol/L) and the presence of urine protein [25, 26].

For MDSi subjects without diabetes, risk appears to be increasing, which is disappointing but not surprising, given the scarcity of the intervention. Occasional lifestyle counseling would not be expected to make a difference. Prevention of diabetes has been shown to be possible in individuals with impaired glucose tolerance subjected to intense, expensive, and supervised lifestyle modification interventions [27, 28]. Despite MDSi's efforts to integrate care with local resources, these services simply do not exist in rural Alberta, where there is a shortage of generalist and specialist physicians [29] and where public health and prevention efforts in the area of chronic disease are only slowly getting underway [1]. 

A project evaluation for the operational period 2004–2006 included analysis of the cost of providing an MDSi service, including startup costs (purchase of diagnostic equipment, vans, staff training, etc.) and variable ongoing costs (salaries and benefits, travel, lab supplies, clinical database development, data storage, etc.) [30]. This analysis estimated a per client startup cost of $166.76 (Canadian dollars) and per client ongoing cost of $721.00, for a total per client cost of $887.76. This compares to a cost analysis completed in 2004 for the SLICK project, which estimated a per client ongoing cost of $734.34 (excluding startup costs) and total per client cost of $917.00 (including startup costs). 

The SLICK analysis further included a cost minimization exercise, comparing SLICK service costs with costs for the same service delivered conventionally via the health care system. Only those costs that could be directly compared (materials, personnel, travel, etc.) were assessed. A SLICK service was estimated to cost $356.55 versus $504.89 for the same service delivered by the health system [31]. Though no similar cost minimization analysis has been completed for MDSi, it is reasonable to assume the cost would approximate that of a SLICK service ($356.55).

## 5. Conclusion

In collaboration with the communities and other stakeholders, we have been able to implement a large-scale clinical and educational diabetes and cardiovascular screening program in rural and remote northern Alberta. MDSi has had to mediate not only geographic, but cultural barriers to service delivery. We have observed significant improvements over time in all measures for subjects with known diabetes, and improvements in A1c and blood pressure measurements for clients without known diabetes. 

During the summers of 2006, 2007, and 2008, we undertook a telephone survey, (though using a different sample of our subjects) to examine whether subjects followup with the recommendation (lifestyle changes, referral for physician care, etc.) MDSi provided them at screening. Overall 51% of subjects surveyed reported visiting a physician after MDSi screening, and (encouragingly) 66% of those MDSi told they had probable diabetes visited a physician. Most subjects surveyed reported they felt a need to change their lifestyle habits to improve their overall health, and the majority of subjects surveyed reported some improvement in at least one lifestyle factor [32]. 

The observed improvements from our present study suggest that MDSi's model may effectively mediate some barriers and support subjects in managing their health. Our emerging evidence supports the rationale for the delivery of screening, prevention and management at the community-level.

## Figures and Tables

**Table 1:** Baseline health indicators. Values are means (±SD) or prevalences (95% CI).

Variables	With known diabetes	Without known diabetes	Total
	(*N* = 180)	(*N* = 629)	(*N* = 809)
BMI (kg/m^2^)	33.4 ± 6.2	31.0 ± 6.3	31.5 ± 6.3
% Overweight (25–29.9)	29.5% (17.0–42.0)	32.0% (25.6–38.4)	31.4% (25.7–37.1)
% Obese (≥30)	67.1% (58.6–75.7)	52.8% (47.4–58.2)	55.9% (51.3–60.5)

Waist circumference (cm)	108.6 ± 13.9	101.6 ± 14.7	103.2 ± 14.8
% Abnormal (≥102 males; ≥88 females)	95.9% (92.9–98.9)	72.8% (68.7–76.9)	78.7% (75.5–81.9)

A1c (%)	7.3 ± 1.6	5.5 ± 0.8	N A
% Poor glucose control (≥7%)	50.0% (41.3–58.7)	1.6% (0.1–3.1)	N A

Systolic BP (mmHg)	136.9 ± 19.0	123.8 ± 21.4	126.7 ± 20.3
Diastolic BP (mmHg)	77.4 ± 9.8	75.3 ± 10.4	75.8 ± 10.3
MAP (mmHg)	97.3 ± 12.1	91.5 ± 12.6	92.7 ± 12.4
% Hypertensive (≥130/80			
with diabetes; 140/90 without	69.5% (61.3–77.7)	21.8% (14.9–28.7)	32.3% (26.6–38.0)
diabetes)			

Total cholesterol (mM)	4.7 ± 1.1	4.9 ± 1.1	4.8 ± 1.1
% Hypercholesterolemia (≥5.24)	30.8% (18.4–43.2)	34.6% (28.3–40.9)	33.8% (28.1–39.5)
